# Factors Limiting Access to Surgical Treatment of Focal Epilepsy in Kazakh Population

**DOI:** 10.3390/ijerph23030343

**Published:** 2026-03-09

**Authors:** Mukhit Dossov, Balzhan Kassiyeva, Nazira Bekenova, Assel Baibussinova, Tamara Vochshenkova, Alisher Aitkaliyev, Akmaral Suleimenova, Aigul Kaptagayeva

**Affiliations:** 1 Medical Center Hospital of the President’s Affairs Administration of the Republic of Kazakhstan, Mangilik El 80, 010000 Astana, Kazakhstan; dossovmukhit@gmail.com (M.D.); nazira.bekenova@mail.ru (N.B.); vochshenkova@gmail.com (T.V.); aitkaliyev1998@gmail.com (A.A.); sulejmenovarajana5@gmail.com (A.S.); 2 Department of Epidemiology and Biostatistics, Semey Medical University, Abai Street 103, 071400 Semey, Kazakhstan; assel_bb@mail.ru; 3 “AIMED” LLP, Kabanbay Batyr Ave 17, 010000 Astana, Kazakhstan; a.kaptagaeva68@gmail.com

**Keywords:** focal epilepsy, epilepsy management, people with Epilepsy, access to care, epilepsy surgery

## Abstract

**Highlights:**

**Public health relevance—How does this work relate to a public health issue?**

- Significant differences by marital status, social protection, and region indicate that social and geographic factors influence who receives surgical treatment, highlighting disparities in access to specialized care for people with drug-resistant focal epilepsy.
- Compared with non-operated patients, individuals with focal epilepsy who underwent surgery had a younger age at seizure onset, a longer duration of epilepsy, and earlier initiation of antiepileptic therapy, suggesting differences in referral timing and continuity of care that may influence the likelihood of receiving surgery.

**Public health significance—Why is this work of significance to public health?**

- Among patients with drug-resistant focal epilepsy identified as candidates for surgical treatment at the Epilepsy Center, only 38.68% underwent surgery. The work highlights the need to adopt a comprehensive approach to epilepsy management that preserves the work capacity and social participation of individuals with focal epilepsy.
- The finding that marital status, rural residence, employment, and especially region strongly influence the likelihood of receiving surgery shows that access to epilepsy treatment is shaped by social and geographic factors rather than medical need alone—indicating disparities that public health systems should address.

**Public health implications—what are the key implications or messages for practitioners, policy makers and/or researchers in public health?**

- The need for prospective analysis and proactive measures in countries with rapidly developing technological capabilities and increasing barriers to the effective management of focal epilepsy.
- The need to accumulate personalized data from patients with focal epilepsy to support the development of technologies capable of predicting and modifying the course of the disease.

**Abstract:**

**Background/Objectives:** Effective management represents a real opportunity to reduce the economic burden of focal epilepsy, which leads to the withdrawal of at least 0.7% of the working-age population from the labor force. The aim of this retrospective observational cross-sectional study is to identify barriers that limit access to surgical treatment for epilepsy among patients with focal epilepsy in Kazakh population. **Methods:** Medical reports from epileptologists on 3112 patients of the Epilepsy Center (Astana) in 2024 were reviewed. The study included original information on 1361 patients with a confirmed diagnosis of focal epilepsy, in accordance with ICD-10 codes G40.0–G40.2. **Results:** Timely detection of focal epilepsy was not associated with socio-demographic or regional factors in our sample. Logistic regression analysis showed that sex and social status did not affect whether patients underwent surgery. However, marital status, employment, and region were significant factors. Married and employed patients had higher odds of remaining non-operated. Among candidates for surgical treatment, 38.68% underwent surgery. **Conclusions:** The markedly higher odds of remaining non-operated outside Astana point to gaps in referral pathways and service availability, emphasizing the need for a comprehensive approach to managing focal epilepsy in countries undergoing rapid technological development.

## 1. Introduction

Epilepsy is one of the most common neurological disorders, with a higher prevalence in low- and middle-income countries [1]. As barriers to epilepsy management exist in most countries [1,2,3], effective management represents a real opportunity to reduce the economic burden caused by the withdrawal from the workforce of at least 0.7% of the working-age population. As a country’s economy develops, alongside declining birth rates and increasing life expectancy, the capacity for effective epilepsy management becomes increasingly important [4].

At the first level of care, within primary healthcare, epilepsy is identified, and symptomatic seizure control is provided using antiseizure medications (ASMs) [5]. Limited resources at the primary level create a gap between the number of people with epilepsy and the number receiving treatment.

Between 1990 and 2021, the age-standardized global prevalence of epilepsy increased by a total of 10.8%, primarily due to focal epilepsy (FE) [1]. FE has an unfavorable clinical prognosis because of decreased sensitivity to ASMs, even with adequate therapy. Early detection of FE, precise localization of its focus, and differentiated use of surgical techniques can improve patients’ quality of life. Initial diagnosis and selection of ASMs are carried out at the secondary level, within specialized medical care. At this level, there is the greatest potential to prevent FE through targeted improvements in perinatal care, prevention and treatment of traumatic brain injury and its consequences, and infectious and parasitic diseases.

In cases of drug-resistant epilepsy (DRE), resection surgery surpasses medical therapy and can lead to seizure freedom. In middle- and high-income countries, a third level of care exists—management of DRE using surgical technologies. As surgical techniques advance, they are becoming increasingly precise, effective, and accessible; non-resective technologies are increasingly emerging as alternatives to resective procedures [6]. Nevertheless, epilepsy surgery remains one of the most underutilized evidence-based interventions in modern medicine [7].

The first two levels of FE management provide access to diagnosis and ASMs, while the third offers access to surgical treatment. Their interaction determines the effectiveness of FE management [8,9]. Expansion of the care levels creates additional challenges in managing FE and requires new solutions. For example, in Japan, access to surgical interventions for DRE was twice as low as in the United States. Establishing a high-technology specialized center that integrates the levels of care and employs a multidisciplinary team approach to FE management improved the situation [10].

Kazakhstan is characterized by a rapidly developing economy. It is expected that by 2030, the country will enter the group of high-income economies. A literature search revealed a limited number of studies on FE management among residents of Central Asia [11,12]. Surgical treatment of FE in Kazakhstan is carried out in three neurosurgical centers and is funded by the state. Their results are comparable to international figures, which may indicate the presence of common pre-surgical challenges [13]. Researchers have noted the negative impact of delays in diagnosis and treatment; however, the potential barriers in Central Asia have not been studied.

A unique feature of the Epilepsy Center of the Medical Center Hospital of the President’s affairs Administration of the Republic of Kazakhstan (hereinafter, Epilepsy Center) is that its multidisciplinary team of highly qualified specialists provides a complete and continuous range of services throughout the entire cycle of diagnosis and treatment of FE in adults—from the initial consultation to completion of the postoperative follow-up period. The Epilepsy Center performs more than 60% of all FE surgical procedures in Kazakhstan. Therefore, the aim of the study was to identify barriers limiting access to surgical treatment for drug-resistant focal epilepsy among the Kazakh population.

## 2. Material and Methods

### 2.1. Study Design and Data Selection

This was a retrospective observational cross-sectional study of the medical records of adult patients who visited the Epilepsy Center in 2024. All 3112 epileptologists’ medical reports were extracted from the Epilepsy Center’s computerized medical information management system. The extraction period spanned from 1 January 2024 to 31 December 2024. To ensure specificity, the data were additionally reviewed by two epileptologists for consistency with the diagnosis and inclusion/exclusion criteria. Individual participant data included date of birth, sex, ethnicity, age at diagnosis, age at initiation of ASM therapy, and duration of FE and drug resistance. For cases of surgical treatment, the age at surgery and the extent of the procedure were recorded. Information on the patient’s social status (employment, receipt of social benefits, marital status), place of residence (city or village), and distance from the Epilepsy Center was also included.

All regions of residence were categorized into four groups based on distance from the Epilepsy Center: Group 1—Astana city (Country capital); Group 2—outside Astana up to 500 km; Group 3—outside Astana 500–999 km; Group 4—outside Astana 1000 km or more. The division of regions based on the length of road routes connected to Astana is driven by the advantages of road transport in Kazakhstan, one of the least densely populated countries in the world, with a population density of about 7.25–8 people per square kilometer.

**Figure 1:**
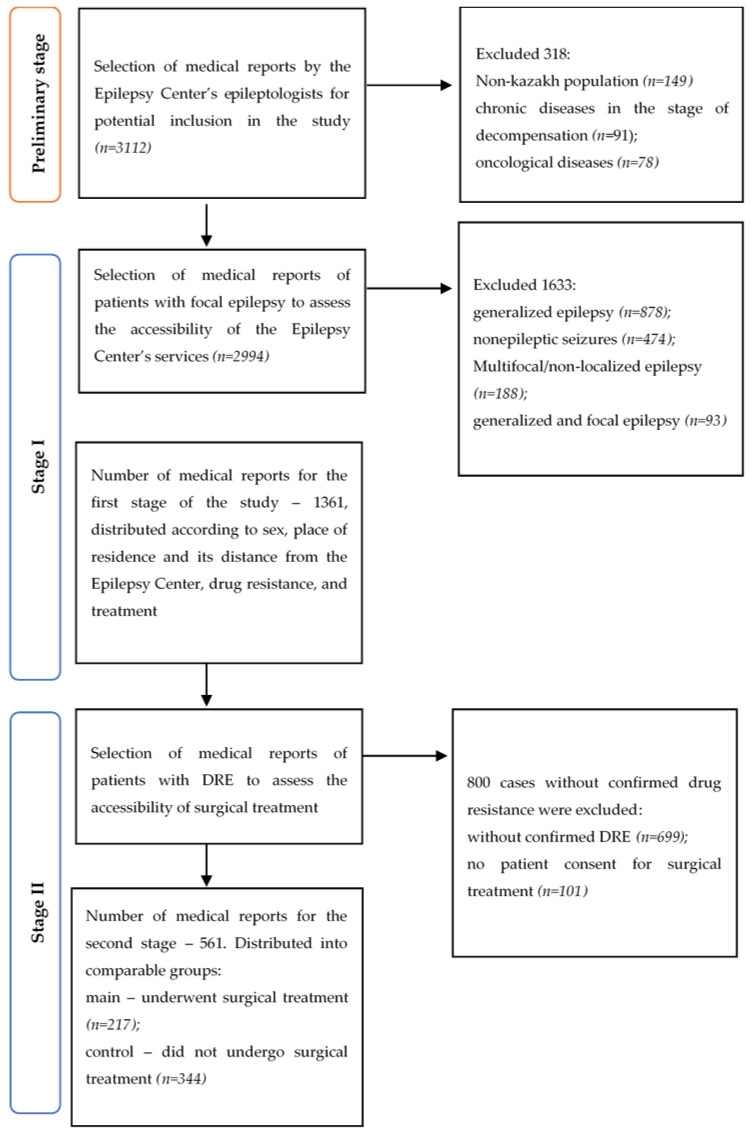
The procedure for selecting unique medical data on patients of the Epilepsy Center in 2024.

The study was conducted in three stages (Figure 1). At the preliminary stage, exclusion criteria included non-Kazakh ethnicity, age under 18, decompensated chronic diseases, and oncological diseases. The preliminary number of reports suitable for further analysis was 2994 cases.

In the first stage, the medical reports of the Epilepsy Center for patients with FE were evaluated. Therefore, the inclusion criteria were limited to FE types consistent with the recommendations of the International League Against Epilepsy (ILAE, 2017) [14]. FE was defined as a form in which seizures originate from a restricted and clearly localized area of the brain with increased paroxysmal activity. A total of 1633 cases not eligible for surgical treatment were excluded (Figure 1).

In the second stage, the medical reports regarding the necessity or performance of surgical treatment for patients with drug-resistant focal epilepsy were evaluated (Figure 1). Inclusion criteria were further restricted to the presence of focal epilepsy in accordance with the ILAE 2017 recommendations [14], confirmation of drug-resistant status, and patient consent for surgical treatment. Drug-resistant FE was defined as failure to achieve sustained seizure remission despite the use of two well-tolerated, appropriately selected, and adequately dosed antiseizure medications, either as monotherapy or in combination. In total, 561 medical reports were analyzed at the second stage across two cohort groups. The main group included 217 reports containing information on a resective surgery, regardless of the place or timing of the procedure.

The control group included 344 reports from an interdisciplinary specialist consortium recognizing the patients as potential candidates for surgical treatment based on the results of preoperative evaluation (electroclinical seizure correlations using video-electroencephalographic monitoring (Neurofax EEG 1200K Nihon Kohden, Tokyo, Japan), structural and functional localization with 3-Tesla MRI (MAGNETOM Skyra eco, Siemens Healthineers, Erlangen, Germany), and, if necessary, assessment of metabolic activity in areas of functional deficit using positron emission tomography (GE Discovery MI Gen 2 PET-CT, Chicago, IL, USA) with 18F-fluorodeoxyglucose, as well as invasive video-electroencephalographic monitoring.

### 2.2. Statistical Analysis

Quantitative data with non-normal distribution were analyzed using the non-parametric Mann–Whitney test for independent groups, and the results are reported as median (Q1; Q3). The normality of the data distribution was assessed using the Shapiro–Wilks criterion. Dichotomous and categorical variables were analyzed using the Chi-square test. A significance level of *p* < 0.05 was considered for determining statistically significant differences.

The association of sociodemographic factors with the probability of receiving surgical treatment was performed by comparing groups of non-operated and operated patients with DRE using binary logistic regression. Statistical analysis was performed using IBM SPSS Statistics 26.0 (IBM Corp., Armonk, NY, USA).

## 3. Results

### 3.1. Stage I: Assessment of Patient Access to the Epilepsy Center

Among both patients with previously known focal epilepsy and those with first documented diagnosis of focal epilepsy at the Epilepsy Center (newly diagnosed with FE), women predominated. The results of our analysis showed no statistically significant differences by sex (Table 1).

Regarding marital status, men slightly predominated among patients with previously known focal epilepsy, whereas women predominated among patients newly diagnosed with focal epilepsy at the Epilepsy Center. However, these differences were not statistically significant (Table 1).

Urban residents predominated in both groups. A substantial proportion of patients lived in urban rather than rural areas. However, no statistically significant differences were identified.

In both the group of patients with previously known focal epilepsy and the group newly diagnosed at the Epilepsy Center, unemployed/unaffiliated individuals predominated. Our analysis also showed no statistically significant differences according to patients’ employment status.

Our study found that the proportion of socially protected patients was higher than that of socially unprotected patients. However, no statistically significant differences were identified between the groups, as socially protected individuals predominated in both (Table 1).

When examining the frequency of operated versus non-operated patients by region of residence, no regional differences between the groups were found.

The results of our study showed that the median age of patients with focal epilepsy newly diagnosed at the Epilepsy Center was slightly higher than that of patients with previously known focal epilepsy (35.0 and 34.5, respectively). However, these differences were not statistically significant.

The age at seizure onset in the group with newly diagnosed with focal epilepsy at the Epilepsy Center was also slightly older than in the group with previously known epilepsy. These differences were likewise not statistically significant.

The duration of epilepsy in both groups exceeded 20 years. It was higher in the group with previously known focal epilepsy than in the second group, but the difference was not statistically significant.

Antiepileptic therapy in the group with focal epilepsy newly diagnosed at the Epilepsy Center was initiated twice as early as in the group with previously known focal epilepsy, although this difference also did not reach statistical significance.

### 3.2. Stage II: Barriers to Receiving Surgical Treatment for Patients with DRE

At the second stage of the study, barriers to receiving surgical treatment were examined for 561 patients with DRE.

The proportion of men was higher in the group of operated patients, whereas women predominated among non-operated patients. Our analysis showed no statistically significant differences by sex (Table 2).

Among non-operated patients, the number of unmarried individuals was higher than that of married individuals. In contrast, among operated patients, married individuals were more common. There was a statistically significant difference between the groups according to marital status. Place of residence may also be statistically significantly associated with access to resection. The number of non-operated patients among urban residents was lower than that of operated patients, whereas among rural residents, non-operated patients were more common. These differences between the groups were statistically significant (Table 2).

Among employed patients, non-operated patients predominated, while among unemployed patients, operated patients were more common. These differences between the groups were also statistically significant (Table 2).

Our study found that the proportion of socially protected patients was higher than that of socially unprotected patients. With respect to access to surgery, socially protected patients were more common among operated patients, and statistically significant differences between the groups were identified (Table 2).

The results of our study showed that, compared with the city of Astana, the number of operated patients in other regions was much lower than that in Astana. Regional differences between operated and non-operated patients were statistically significant and were reflected in the predominance of residents of Astana among the operated patients (Table 2).

Our study also showed that the median age of non-operated patients was slightly higher than that of operated patients. The age at seizure onset was younger in operated patients than that in non-operated patients. These differences were statistically significant (Table 2). However, the duration of epilepsy was statistically significantly longer in the group of operated patients compared with non-operated patients. Antiepileptic therapy in the group of operated patients was initiated more than four times earlier than in the group of non-operated patients.

The results of the logistic regression analysis showed that sex does not influence whether a patient undergoes surgery (Table 3). However, marital status was identified as a factor affecting this outcome. As shown in the table, married patients had twice the odds of being non-operated compared to unmarried patients.

When assessing the effect of place of residence, it was found that unemployed patients had lower odds of being in the non-operated group compared to employed patients. The presence of social status in our sample did not influence whether a patient underwent surgery (Table 3).

Regarding regional differences, the results demonstrated that patients from other regions had substantially higher odds of being non-operated. Compared to Region 1 (Astana), patients from Region 2 had three times higher odds of being non-operated. Patients from Regions 3 and 4 had four times higher odds of being non-operated (Table 3).

## 4. Discussion

The results of our study report that timely detection of FE was not associated with socio-demographic or regional factors in our sample. However, surgical treatment may be associated with patients’ social status, place of residence, and employment status.

Access to healthcare is defined as the ability to obtain necessary medical services when they are needed [15]. Structural barriers refer to differences in access to medical care caused by geographic and financial limitations. Resource constraints in healthcare primarily manifest as limited access to services [16]. The resulting unmet need for specialized medical care is effectively paid for directly by patients at the Epilepsy Center. Consequently, regional issues of a limited-service structure are compounded by low personal income, further restricting patients’ ability to seek care. The timeliness and quality of surgical treatment directly affect its effectiveness [15,16]. In our study, only 38.68% of patients in need received surgical treatment; their mean age was 35 years, compared with 30.6 years in the United States, highlighting the significance of structural barriers to access for FE surgery in Kazakhstan.

Structural barriers reflect the uneven distribution of resources that promote health, a characteristic of all countries [16]. Existing independently of the patient, these barriers initiate and exacerbate the effects of other obstacles.

Healthcare-related barriers are constraints in access for both physicians and patients to resources necessary for effective epilepsy management.

In the medical records reviewed, it was noted that therapeutic monitoring of ASMs had not been performed at previous levels of FE management. In middle- and upper-middle-income countries, monitoring is rarely used and is primarily applied to assess ASM toxicity [17,18]. In high-income countries, however, it is an important complement to clinical practice when initiating or adjusting drug therapy, as well as in cases of DRE. The high cost of monitoring in these countries is offset by treatment effectiveness, adherence, and increased patient trust [2]. Only 2 out of 1361 medical records contained a single result from neuropsychological testing. In high-income countries, such testing is most applied at early stages of diagnosis and treatment, as well as in employment-related limitations [2]. The combined use of drug and neuropsychological monitoring in treatment could reduce the impact of structural barriers that limit employment opportunities and contribute to high dependency among FE patients.

The limited range of surgical interventions for FE in Kazakhstan, restricted to resective and palliative methods, represents another healthcare-related barrier. In high-income countries, the spectrum of surgical technologies has expanded to include minimally invasive approaches such as laser interstitial thermal therapy, radiofrequency ablation, stereotactic radiosurgery, and focused ultrasound [2,19].

It is worth noting that among healthcare system-related barriers is the fragmented management of FE in Kazakhstan. Communication between neurologists at the level of specialized medical care and the Epilepsy Center is not regulated by official guidelines. As a result, patients often seek care at the Epilepsy Center independently and personally pay for diagnostic services that should have been provided at the level of specialized medical care. Only 38.68% of patients eligible for surgical treatment received it, partly due to insufficient state funding for surgical care at the Epilepsy Center. Integrating medical practice into a unified FE management strategy could mitigate the negative effects of structural barriers, while accurate statistics at the level of specialized medical care would ensure an adequate volume of surgical interventions.

Another observation from our study is that among operated patients, only 13.36% were older than 45 years. We could speculate that age as a barrier to surgical treatment in Kazakhstan might be influenced by stigma due to possible prejudices (e.g., perceived high risk of adverse outcomes, doubts related to multifocal disorders). Stigma, affecting behavior toward one’s health [20], is a widely recognized obstacle and is common among patients with epilepsy [21,22].

Physiological barriers include differences in access to molecular-genetic technologies capable of preventing or altering the course of disease. To date, these technologies remain largely inaccessible even in high-income countries. Nevertheless, they represent a potential new line of epilepsy management that could eventually replace current practice. A country’s readiness to adopt these global advances will depend on the integration of existing management lines [5]. The most relevant physiological barrier for Kazakhstan is the management of comorbid conditions. For example, individuals with depression have a sevenfold higher risk of developing epilepsy due to bidirectional links, while people with epilepsy are at increased risk of developing depression [23]. Access to neuropsychological testing could enable more effective management of these comorbidities and mitigate the negative effects of both physiological and healthcare-system barriers.

One of the main limitations of the study is that although social protection, employment, and marital status were assessed, direct measures of income, education, or healthcare costs were not included, limiting the ability to fully analyze financial barriers. The study was conducted at one epilepsy center, which may limit the generalizability of the findings to other regions or healthcare settings in Kazakhstan. Moreover, our study included only patients of Kazakh ethnicity, excluding non-Kazakh patients. This selection was made to reduce potential confounding effects of sociocultural differences between ethnic groups within Kazakhstan. However, this introduces a selection bias that limits the generalizability of our findings to the entire population. Consequently, our analysis may not fully capture sociocultural barriers experienced by non-Kazakh patients, and the identified associations between sociodemographic factors and access to surgery may differ in other ethnic groups. Future studies should include multiethnic samples to comprehensively assess access to epilepsy care across all population groups.

## 5. Conclusions

The study concludes that, in Kazakhstan—as in other countries—barriers exist that limit access to surgical treatment for focal epilepsy. Strengthened integration and coordination across the different levels of focal epilepsy care may improve treatment effectiveness despite substantial structural constraints.

Evidence-based, coordinated measures can ensure that patients with FE have maximal access to high-quality medical care in real-world conditions and help preserve their work capacity. Only under such circumstances can an economic benefit from FE management be expected in countries with rapidly developing technological capabilities.

## Figures and Tables

**Table 1:** Social differences among FE and newly diagnosed FE patients at the Epilepsy Center.

Factors	FE Cases	Newly Diagnosed at the Epilepsy Center	*p*-Value
Sex	800	560	0.25
Male	371 (46.4%)	242 (43.2%)	
Female	429 (53.6%)	318 (56.8%)	
Age (Me, (Q1–Q3))	34.5 (29.0–39.0)	35.0 (32.0–45.0)	0.12
Age of FE Onset (Me, (Q1–Q3))	13.0 (6.0–12.0)	14.0 (7.0–23.0)	0.13
Duration of FE (Me, (Q1–Q3))	22.0 (13.0–27.0)	21.0 (11.0–28.0)	0.79
Start of ASM Therapy (Me, (Q1–Q3))	3.0 (1.0–18.8)	6.0 (1.0–24.0)	0.15
Marital Status	800	560	0.39
Married	412 (51.5%)	275 (49.1%)	
Unmarried	388 (48.5%)	285 (50.9%)	
Place of Residence	800	560	0.25
Urban	667 (83.4%)	480 (85.7%)	
Rural	133 (16.6%)	80 (14.3%)	
Employment	800	560	0.58
Employed	296 (37.0%)	205 (36.6%)	
Unemployed	504 (63.0%)	355 (63.4%)	
Social Support	800	560	0.64
Yes	520 (65.0%)	370 (66.1%)	
No	280 (35.0%)	190 (33.9%)	
Region	800	560	0.24
Astana	371 (46.4%)	247 (44.1%)	
Other regions	429 (53.6%)	313 (55.9%)	

Abbreviations: FE cases—focal epilepsy cases; Me, (Q1–Q3))—Median, (Q1–Q3)).

**Table 2:** Social differences among DRE patients at the Epilepsy Center depending on access to surgical treatment.

Factors	Non-Operated	Operated	*p*-Value
Sex	344	217	0.37
Male	169 (49.1%)	115 (52.9%)	
Female	175 (50.9%)	102 (47.1%)	
Age (Me, (Q1–Q3))	39.0 (34.0–46.8)	35.0 (30.0–41.0)	<0.0001
Age of FE Onset (Me, (Q1–Q3))	15.0 (9.0–21.8)	13.0 (6.0–19.5)	0.02
Duration of FE (Me, (Q1–Q3))	13.0 (6.0–22.0)	24.0 (19.0–30.0)	0.01
Start of ASM Therapy (Me, (Q1–Q3))	22.0 (17.0–28.0)	5.0 (1.0–20.5)	<0.0001
Marital Status	344	217	<0.0001
Married	142 (41.3%)	148 (68.2%)	
Unmarried	202 (58.7%)	69 (31.8%)	
Place of Residence	344	217	<0.0001
Urban	276 (80.2%)	211 (97.2%)	
Rural	68 (19.8%)	6 (2.8%)	
Employment	344	217	0.001
Employed	132 (38.4%)	55 (25.3%)	
Unemployed	212 (61.6%)	162(74.7%)	
Social Support	344	217	0.45
Yes	231 (67.2%)	139 (64.1%)	
No	113 (32.8%)	78 (35.9%)	
Region	344	217	<0.0001
Astana	113 (32.9%)	167 (76.9%)	
Other regions	231 (67.2%)	50 (23.1%)	

Abbreviations: FE cases—focal epilepsy cases; Me, (Q1–Q3)—Median, (Q1–Q3).

**Table 3:** The influence of sociodemographic factors on the probability of receiving surgical treatment.

Factors	Non-Operated	Operated	OR (95% CI)	*p*-Value
Male	169 (49.1%)	115 (52.9%)	1	0.60
Female	175 (50.9%)	102 (47.1%)	0.90 (0.60–1.35)	
Married	142 (41.3%)	148 (68.2%)	2.28 (1.51–3.46)	<0.0001
Unmarried	202 (58.7%)	69 (31.8%)	1	
Urban	276 (80.2%)	211 (97.2%)	2.35 (0.92–6.03)	0.075
Rural	68 (19.8%)	6 (2.8%)	1	
Employed	132 (38.4%)	55 (25.3%)	1	<0.003
Unemployed	212 (61.6%)	162(74.7%)	0.49 (0.31–0.71)	
Social Support Yes	231 (67.2%)	139 (64.1%)	1	0.70
Social Support No	113 (32.8%)	78 (35.9%)	1.09 (0.69–1.72)	
Region	344	217	-	-
Region 1 Astana	112 (32.6%)	168 (77.4%)	1	-
Region 2 (500 m)	69 (20.1%)	28 (12.9%)	3.14 (1.83–5.37)	<0.0001
Region 3 (500–1000)	41 (11.9%)	12 (5.5%)	4.05 (1.93–8.50)	<0.0001
Region 4 (>1000)	122 (35.5%)	9 (4.14%)	3.93 (1.45–10.6)	0.007

Abbreviations: OR (95% CI)—odds ratio (95% Confidence Interval).

## Data Availability

The data presented in this study are available on request from the corresponding author due to the protection of primary data.
